# Range-wide phenotypic and genetic differentiation in wild sunflower

**DOI:** 10.1186/s12870-016-0937-7

**Published:** 2016-11-10

**Authors:** Edward V. McAssey, Jonathan Corbi, John M. Burke

**Affiliations:** 1 Department of Plant Biology, University of Georgia, Miller Plant Sciences Building, Athens, GA 30602 USA; 2 University of Georgia, Center for Applied Genetic Technologies, 111 Riverbend Road, Athens, GA 30602 USA; 3 Université de Lyon, F-69000, Lyon; Université Lyon 1; CNRS, UMR5558, Laboratoire de Biométrie et Biologie Evolutive, F-69622 Villeurbanne, France

**Keywords:** Latitudinal variation, Local adaptation, Phenotypic differentiation, Population genetics, Sunflower

## Abstract

**Background:**

Divergent phenotypes and genotypes are key signals for identifying the targets of natural selection in locally adapted populations. Here, we used a combination of common garden phenotyping for a variety of growth, plant architecture, and seed traits, along with single-nucleotide polymorphism (SNP) genotyping to characterize range-wide patterns of diversity in 15 populations of wild sunflower (*Helianthus annuus* L.) sampled along a latitudinal gradient in central North America. We analyzed geographic patterns of phenotypic diversity, quantified levels of within-population SNP diversity, and also determined the extent of population structure across the range of this species. We then used these data to identify significantly over-differentiated loci as indicators of genomic regions that likely contribute to local adaptation.

**Results:**

Traits including flowering time, plant height, and seed oil composition (i.e., percentage of saturated fatty acids) were significantly correlated with latitude, and thus differentiated northern vs. southern populations. Average pairwise F_ST_ was found to be 0.21, and a STRUCTURE analysis identified two significant clusters that largely separated northern and southern individuals. The significant F_ST_ outliers included a SNP in *HaFT2*, a flowering time gene that has been previously shown to co-localize with flowering time QTL, and which exhibits a known cline in gene expression.

**Conclusions:**

Latitudinal differentiation in both phenotypic traits and SNP allele frequencies is observed across wild sunflower populations in central North America. Such differentiation may play an important adaptive role across the range of this species, and could facilitate adaptation to a changing climate.

**Electronic supplementary material:**

The online version of this article (doi:10.1186/s12870-016-0937-7) contains supplementary material, which is available to authorized users.

## Background

Local adaptation, wherein populations have higher fitness in their ‘home’ environments than in non-native locales, is a topic of great interest in the field of evolutionary biology (e.g., [1]). The genetic basis of such adaptive divergence has not, however, been elucidated in the vast majority of non-model organisms. For plants, the selective pressures leading to local adaptation can include a variety of abiotic and biotic factors such as: soil type [2–4], water availability [5], photoperiod [6], temperature [7], herbivores [8], mycorrhizal associations [9], and proximity to agricultural fields [10]. Because these selective pressures are expected to produce characteristic patterns of genetic variation in and near genes conferring adaptive differences, population genetic approaches have the potential to provide insight into the genes, or at least genomic regions, responsible for producing locally adapted traits across the range of a species.

In the case of divergent selection, which would be expected to play an important role in the production of locally adapted populations, the focus is typically on measures of population genetic differentiation. More specifically, divergent selective pressures would be expected to produce elevated population structure in the vicinity of targeted genes relative to the genome-wide average (e.g., [11–14]). In contrast, balancing selection would be expected to result in much lower levels of population genetic differentiation [15, 16]. When combined with high-throughput genotyping approaches, such population genetic approaches have been used to identify genes thought to be involved in adaptation in a variety of species, including boreal black spruce [17], Atlantic cod [18], prairie-chickens [19], and moor frogs [20].

In addition to overall levels of population differentiation, clinal patterns of genetic variation can also be indicative of local adaptation (e.g., [21, 22]). A variety of environmental variables typically vary across the ranges of species, and thus there may be selection for different phenotypic values at the extremes of a species’ range. While allele frequencies at many loci might exhibit weak correlations across a given environmental contrast due to the joint effects of genetic drift and gene flow, alleles at loci that play an important role in local adaptation should clearly correlate with relevant environmental variables [21]. For example, adaptive clines in allele frequency have been identified in *Arabidopsis thaliana* for the flowering time genes *FRIGIDA* [23] and *PHYTOCROME C* [24], in *Populus tremula* for the flowering time gene *PHYTOCHROME B2* [25], in *Drosophila melanogaster* for the insulin-signaling gene *INSULIN-LIKE RECEPTOR* [26], and in *Peromyscus polionotus* for the coat color gene *AGOUTI* [27]. While the above studies have provided tremendous insight into the genetic basis of local adaptation, studies of non-model organisms will help to broaden our understanding of this fundamental evolutionary process. In the present paper, we report on range-wide patterns of phenotypic and genetic diversity in common sunflower, *Helianthus annuus*.

Sunflower is a member of the Compositae (a.k.a., the Asteraceae), which is one of the largest and most diverse families of flowering plants. The native range of common sunflower spans much of North America, and wild populations occur in habitats that are characterized by variation in a wide range of environmental variables, including: photoperiod, growing season, minimum/maximum temperatures, and precipitation. Common sunflower is also the wild progenitor of cultivated sunflower (also *H. annuus*), which is native to east-central North America [28–30] and is one of the world’s most important oilseed crops [31]. Cultivated sunflower shows significant phenotypic differences as compared to common sunflower, including branching, flowering time, plant height, and various seed traits [32].

Here, we describe patterns of phenotypic and genetic diversity within and among 15 wild sunflower populations across a latitudinal gradient in central North America. We grew and phenotyped individuals from these populations in a greenhouse environment and genotyped them using a single-nucleotide polymorphism (SNP) array targeting 384 loci distributed throughout the sunflower genome. We used these data to investigate geographic patterns of phenotypic differentiation, describe overall patterns of population genetic variation, and identify loci that harbor the population genetic signature of local adaptation. We also placed our population genetic results in the context of prior quantitative trait locus (QTL) mapping studies in sunflower to determine whether highly differentiated loci co-localize with known QTL regions.

## Methods

### Plant materials and phenotypic analyses

**Fig. 1:**
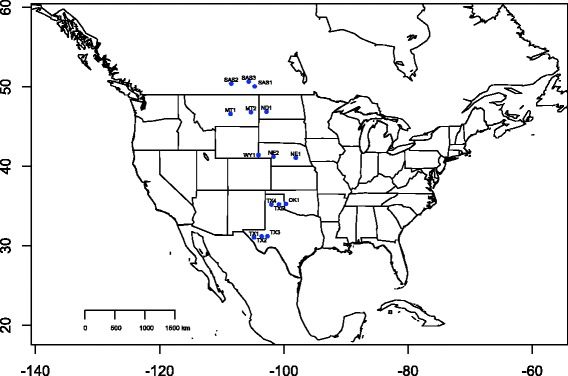
Map of the locations of the 15 populations used in this study in the central USA and Canada. Map was constructed in R using the library ‘maps’ [65]

Seeds from 15 wild-collected populations of *H. annuus* were obtained from the USDA’s North Central Regional Plant Introduction Station (Ames, IA). These populations, which were sampled from a range of latitudes across central North America (Fig. 1; Table 1), were selected to represent truly wild populations that appear to be free from the effects of past introgression with cultivated sunflower (L. Marek and G. Seiler, personal communication). Care was taken to avoid sampling different subspecies of *H. annuus* (e.g., *H. annuus* ssp. *Texanus*), as that could inflate genetic structure and/or phenotypic differentiation. Prior to germination, all seeds were cleaned with 3 % hydrogen peroxide, rinsed with deionized water, and placed on moist filter paper in a petri dish. To break dormancy, petri dishes were placed at 4 C in a dark cold room for 14 days. After the cold treatment, they were moved into a growth room where they were maintained under 16 h days at 23 C. Following germination, seedlings were planted in soil trays. Once established, these seedlings were transplanted into soil pots (900 Classic, Nursery Supplies Inc, Kissimmee, FL) and moved to the greenhouse, where supplemental lighting provided a consistent cycle of 16 h days and 8 h nights.

**Table 1:** Range-wide population sampling information

Population	State/Province	Country	USDA PI	Sample Size	Latitude	Longitude
TX1	Texas	USA	413160	20	31.041	−104.821
TX2	Texas	USA	664692	20	31.189	−103.578
TX3	Texas	USA	468476	20	31.206	−102.635
TX4	Texas	USA	468477	20	35.190	−102.010
TX5	Texas	USA	468482	20	35.199	−100.799
OK1	Oklahoma	USA	468486	12	35.262	−99.669
NE1	Nebraska	USA	586870	20	41.063	−98.091
NE2	Nebraska	USA	659440	20	41.211	−101.649
WY1	Wyoming	USA	649806	20	41.418	−104.098
MT1	Montana	USA	531035	20	46.585	−108.592
MT2	Montana	USA	586817	20	46.795	−105.302
ND1	North Dakota	USA	613750	20	46.879	−102.789
SAS1	Saskatchewan	Canada	592320	19	50.048	−104.707
SAS2	Saskatchewan	Canada	592311	15	50.394	−108.480
SAS3	Saskatchewan	Canada	592316	20	50.660	−105.665

Plants were arranged in the greenhouse in four blocks, each of which contained five individuals from each of the 15 populations (75 individuals total per block). All plants were phenotyped for a variety of traits, including: days to four pairs of true leaves, days to flowering, plant height at senescence, branching architecture, seed size, and seed oil content/composition. Because wild sunflower is self-incompatible, manual crosses were performed to produce seeds. This involved intercrossing individuals within populations (i.e., bulked pollen collected from individuals within a population was used to pollinate individuals within that population), with inflorescences being bagged to prevent cross-contamination. Seeds were then collected at physiological maturity and phenotyped. Oil traits were assessed following established protocols [32]. Briefly, percent oil content was determined via pulsed nuclear magnetic resonance (NMR) analyses using a Bruker MQ20 Minispec NMR analyzer (Billerica, MA) that had been calibrated with known standards. Fatty acid composition was determined by gas chromatography (Hewlett-Packard, Palo Alto, CA) with known fatty acid standards (Nu-Check Prep, Elysian, MN).

All traits were tested for deviations from normality by determining whether a frequency histogram of trait values across all 286 full grown individuals (14 of the originally planted individuals died early in development, but at least 12 individuals for each population were analyzed (Table 1)) was significantly different from a normal distribution with the Shapiro-Wilk test in JMP 11 (SAS Institute, Cary, NC) and trait values were transformed using a Box-Cox transformation [33] as necessary. Restricted maximum likelihood was used with region as a fixed effect (blocks and a block-by-region interaction were included as random effects) to test for regional differences in trait values. For fatty acid traits, the date of fatty acid extraction was used as a blocking factor instead of greenhouse block because an inspection of the raw data indicated clear variation in extraction efficiency across days. Least-squares means were compared amongst regions using Tukey’s test.

### DNA extractions and SNP genotyping

Leaf tissue was harvested from the 286 fully grown (Table 1) individuals described above and DNA was extracted using the Qiagen DNeasy Plant Mini Kit (Valencia, CA). All DNA samples were quantified using a NanoDrop (Thermo Scientific, Wilmington, DE) and diluted to 50 ng/μl prior to genotyping. Each sample was then genotyped using a GoldenGate assay (Illumina, San Diego, CA) targeting 384 SNPs selected from the larger collection of sunflower SNPs described by Bachlava et al., [34]. These loci were chosen to provide even coverage of the 17 sunflower linkage groups (LGs), with an average of one SNP every 3.5 cM. Genotype calls were made using Illumina’s GenomeStudio (ver. 2011.1) followed by manual inspection. Loci that exhibited aberrant hybridization signals (perhaps due to presence/absence variation or the occurrence of duplicate genes), an overall lack of polymorphism (i.e., minor allele frequency < 0.05), and/or large amounts of missing data (i.e., fraction of missing data > 0.05) were removed prior to population genetic analysis. A total of 246 loci (average = 14.5 per LG; range = 11–20 per LG) were retained for further analysis (http://dx.doi.org/10.5061/dryad.6p1c4).

### Population genetic analyses

Measures of genetic diversity, including the percentage of polymorphic loci, observed heterozygosity (H_o_), and Nei’s unbiased expected heterozygosity (UH_e_; [35]) were calculated at the population level using GenAlEx (version 6.501; [36]). We also used GenAlEx to investigate genetic differentiation amongst populations by performing an analysis of molecular variance (AMOVA) with 999 permutations to determine the level of population structure in our dataset. Finally, the program STRUCTURE (version 2.3.4) [37] was used to investigate population genetic structure across the species range. Specifically, STRUCTURE was run using the admixture model from *K* = 1 to 17 population genetic clusters with a burn-in of 100,000 and 1,000,000 MCMC iterations (with 20 replicates for each *K* value). Results were imported into STRUCTURE Harvester [38] where the most likely value of *K* was determined using the Delta*K* method [39]. STRUCTURE, was additionally used to test individual subsets of the data to investigate finer levels of genetic structure.

The potential role of local selective pressures in shaping diversity at individual loci was investigated using multiple approaches. First, we used Arlequin to calculate 20,000 simulations in order to obtain a null distribution for F_ST_, which was then used to develop a 99 % confidence interval for high and low outlier identification (version 3.5.1.2; [13]). In general terms, over-differentiated loci are regarded as candidates for local adaptation, while under-differentiated loci are generally viewed as candidates for balancing selection [15, 16], or possibly a sweep across multiple populations [40]. BayeScan was also used to test for selection by comparing the posterior probabilities of two models (selection vs. no selection) for each locus [14]. Following Foll and Gaggiotti (version 2.1; [14]), loci whose posterior probability for the model including selection was greater than 0.91 were regarded as being ‘strong’ F_ST_ outlier candidates. We then mined the sunflower QTL literature to identify any QTL whose confidence interval co-localized with a putative local adaptation SNP identified in this study, as such overlapping loci might be particularly attractive candidate regions for future research. Co-localization information was obtained using previously published studies from a variety of sunflower crosses [32, 41–44].

## Results

### Phenotypic diversity

We identified numerous traits that exhibited differentiation amongst the five sampled regions, with latitude being a significant factor in the partitioning of phenotypic diversity for traits such as flowering time, plant height, branching, and a number of seed oil traits (Table 2; Additional file 1). Individuals from the southern regions (Texas and Oklahoma, Regions 1 and 2; Table 2; Additional file 1) tended to flower later, grow taller, have thicker stems, and have a higher proportion of saturated fatty acids within their seeds compared to individuals from the northern regions found in Saskatchewan, North Dakota and Montana (Regions 4 and 5; Table 2; Additional file 1). The fatty acid composition data also showed some interesting trends, with the saturated type (i.e., palmitic and stearic acid) showing the same sort of regional differentiation as noted above. In contrast, the unsaturated types (i.e., oleic acid and linoleic acid) did not show significant differences between regions. Seed oil content showed no significant differences among regions across the entire range (Table 2; Additional file 1). Aside from the aforementioned differentiation in saturated fatty acid percentage in seed oils, regions were significantly differentiated for seed length with respect to latitude. While seed weight and seed width both exhibit some regional differences, the differences were not due to latitude as the most southern region was not significantly different from the most northern region for these two traits (Table 2; Additional file 1). Notably, the latitudinal trends found in saturated fatty acid content and flowering time are consistent with the results of previous studies [45, 46]. While total branching exhibited significant differences among regions, there was no clear trend with respect to latitude. However, plants from Texas and Oklahoma (Regions 1 and 2; Table 2; Additional file 1) had significantly more top branching compared to the three northern regions. Other plant architecture traits, such as branch length and the extent of secondary, tertiary, or higher-order branching, were significantly different between regions, but those differences likewise did not show a latitudinal pattern (Table 2; Additional file 1). Interestingly, no traits exhibited significant differentiation between all five regions (Table 2; Additional file 1).

**Table 2:** Phenotypic variability among five latitudinal regional groupings of sunflower populations

Trait	F-ratio	P-value	Region 1: TX1, TX2, TX3	Region 2: TX4, TX5, OK1	Region 3: NE1, NE2, WY1	Region 4: MT1, MT2, ND1	Region5: SAS1, SAS2, SAS3
Days to Four Pairs of Leaves	4.1906	0.0267	23.42^B^	23.85^A,B^	24.17^A,B^	24.28^A,B^	24.98^A^
Days to Flowering	226.6019	<.0001	69.93^B^	75.80^A^	68.77^B^	51.75^C^	49.31^C^
Days between Four Pairs of Leaves and Flowering	74.905	<.0001	46.52^A,B^	51.98^A^	44.53^B^	27.47^C^	30.44^C^
Plant Height (cm)	89.4095	<.0001	191.55^A^	170.32^A,B^	164.19^B^	110.85^C^	78.17^D^
Longest Branch Length (cm)	15.667	0.0002	57.72^A^	41.66^B^	47.83^B^	57.86^A^	45.68^B^
Degree of Branching^1^	18.3082	<.0001	3.02^A^	2.46^B^	2.62^B^	3.30^A^	2.64^B^
Stem Diameter (mm)	17.9575	<.0001	15.13^A^	13.82^A,B^	12.98^B^	13.19^B^	10.46^C^
Total Branching	9.0473	0.0012	10.20^A,B^	8.58^B,C^	7.31^C^	10.97^A^	9.87^A,B^
Top Half Branching	35.1134	<.0001	7.52^A^	7.21^A^	5.17^B^	4.02^B,C^	3.43^C^
Weight per Seed	14.0714	0.0003	0.007^B,C^	0.006^C^	0.008^A,B^	0.009^A^	0.008^A,B^
Seed Length (mm)	31.0011	0.0005	4.81^B,C^	4.64^C^	4.92^B^	5.14^A^	5.27^A^
Seed Width (mm)	6.1076	0.009	2.40^A,B^	2.28^B^	2.41^A,B^	2.55^A^	2.45^A,B^
Percent Palmitic Acid	31.6781	0.0003	5.05^A^	4.60^A,B^	4.09^B^	3.93^B^	3.09^C^
Percent Stearic Acid	13.7987	0.014	2.26^A^	2.34^A,B^	1.56^A,B,C^	1.23^B,C^	0.82^C^
Percent Linoleic Acid	0.6313	0.6587	60.01^A^	52.39^A^	57.94^A^	63.45^A^	59.06^A^
Percent Oleic Acid	0.6725	0.6353	32.69^A^	40.69^A^	36.33^A^	31.39^A^	37.01^A^
Percent Saturated Fatty Acids	60.4987	0.0043	7.30^A^	6.93^A,B^	5.66^B,C^	5.15^C^	3.90^D^
Percent Oil	1.059	0.4418	23.48^A^	21.09^A^	22.98^A^	22.84^A^	23.05^A^

^1^Degree of branching describes the greatest amount of branching attained (i.e., a value of 2 corresponds to secondary, 3 corresponds to tertiary)

Values that share a superscript lette﻿r are not statistically different

### Population genetic structure

**Fig. 2:**
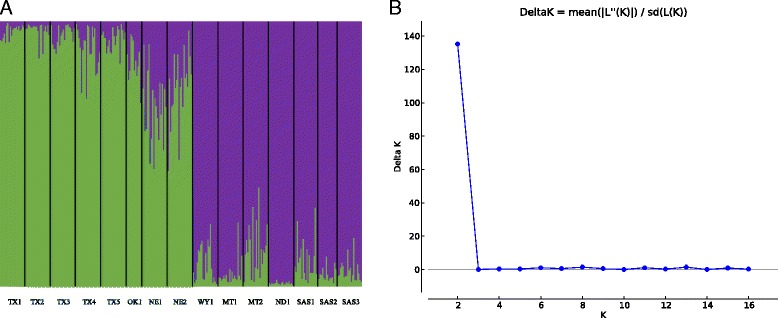
Population genetic structure of wild sunflower individuals. **a** STRUCTURE bar plot of full dataset. Populations correspond to those in Table 1. **b** Delta*K* plotted across all values of *K* tested. Figure constructed in STRUCTURE HARVESTER [38]

Calculation of population genetic statistics for each of the 15 populations revealed a substantial, albeit variable, amount of genetic diversity across the range of wild sunflower (Table 3). There was no trend towards either latitudinal extreme of the range having a reduced level of genetic diversity (Table 3). However, two populations (WY1 and ND1) exhibited a noticeably lower percentage of polymorphic loci compared to the other 13 populations. An analysis of molecular variance revealed that approximately 20 % of the observed genetic variation could be attributed to population level differentiation (data not shown). Of the remaining genetic variation, 76 % was seen at the within individual levels whereas only 4 % was found at the among-individual level. A STRUCTURE [37] analysis of the data coupled with the Delta*K* method for determining the most likely number of population genetic clusters [39] identified *K* = 2 clusters (Fig. 2). The STRUCTURE bar plot for *K* = 2 revealed a north-south divide with the east-central portion of Region 3 corresponding to a transition zone (Fig. 2). An additional STRUCTURE run containing only the southernmost six populations also indicated that *K* = 2. For this level of *K*, TX1 was separated from the remaining five populations found in Texas and Oklahoma, although *K* = 6 showed a secondary peak (Additional files 2, 3, and 4). When the northernmost six populations were analyzed by STRUCTURE, *K* = 2 was again the most well-supported number of genetic groups. Similar to the result for the southern portion of the range, only a single population (ND1) in the northern portion of the range was separated from the other five populations at *K* = 2 (Additional files 5 and 6). Additionally, since the initial full dataset STRUCTURE analysis suggested that two of the three middle latitude populations were more southern while the other population appeared more northern we performed more STRUCTURE analyses to explore differentiation within the middle of the range. To study the middle latitude populations we added NE1 and NE2 to the southern dataset, and WY1 to the northern dataset for further testing. When we performed STRUCTURE analyses of these larger groupings, we found that *K* = 3 for the northern cluster. The three clusters corresponded to ND1, WY1, and the remaining populations. Additionally, we found that *K* = 2 for the southern cluster with the one cluster corresponding to NE2 individuals, and the other contained the remaining seven populations.

**Table 3:** Mean and standard error (SE) of population genetic statistics for 15 wild sunflower populations

Population	USDA PI		Na^a^	Ne^b^	Ho^c^	uHe^d^	F_IS_^e^	P^f^
TX1	413160	Mean	1.85	1.41	0.26	0.25	−0.03	0.85
		SE	0.02	0.02	0.01	0.01	0.02	
TX2	664692	Mean	1.80	1.46	0.26	0.28	0.04	0.80
		SE	0.03	0.02	0.01	0.01	0.02	
TX3	468476	Mean	1.84	1.49	0.28	0.30	0.05	0.84
		SE	0.02	0.02	0.01	0.01	0.02	
TX4	468477	Mean	1.86	1.49	0.28	0.30	0.03	0.86
		SE	0.02	0.02	0.01	0.01	0.02	
TX5	468482	Mean	1.91	1.49	0.26	0.30	0.10	0.91
		SE	0.02	0.02	0.01	0.01	0.02	
OK1	468486	Mean	1.80	1.46	0.26	0.28	0.05	0.80
		SE	0.03	0.02	0.01	0.01	0.02	
NE1	586870	Mean	1.91	1.51	0.30	0.31	0.00	0.91
		SE	0.02	0.02	0.01	0.01	0.02	
NE2	659440	Mean	1.69	1.42	0.24	0.25	0.03	0.69
		SE	0.03	0.02	0.01	0.01	0.02	
WY1	649806	Mean	1.57	1.36	0.22	0.21	−0.05	0.57
		SE	0.03	0.03	0.02	0.01	0.02	
MT1	531035	Mean	1.89	1.46	0.30	0.28	−0.08	0.89
		SE	0.02	0.02	0.01	0.01	0.02	
MT2	586817	Mean	1.86	1.48	0.28	0.29	−0.01	0.86
		SE	0.02	0.02	0.01	0.01	0.02	
ND1	613750	Mean	1.59	1.36	0.24	0.22	−0.12	0.59
		SE	0.03	0.02	0.02	0.01	0.02	
SAS1	592320	Mean	1.89	1.50	0.27	0.30	0.08	0.89
		SE	0.02	0.02	0.01	0.01	0.02	
SAS2	592311	Mean	1.82	1.47	0.28	0.29	0.02	0.82
		SE	0.02	0.02	0.01	0.01	0.02	
SAS3	592316	Mean	1.85	1.48	0.27	0.29	0.04	0.85
		SE	0.02	0.02	0.01	0.01	0.02	

^a^ Number of alleles per locus

^b^ Effective number of alleles per locus

^c^ Observed heterozygosity

^d^ Unbiased expected heterozygosity

^e^ Inbreeding coefficient

^f^ Percent polymorphic loci

### Outlier identification

Multiple outlier identification programs highlighted the existence of an overlapping set of loci that exhibit the signature of local adaptation (Table 4). Arlequin identified eight loci that were highly differentiated in a global F_ST_ calculation (all possible pairwise F_ST_ combinations; 99 % confidence intervals). These loci included: one SNP on LG4 with no annotation; two SNPs located near the distal end of LG 6, one in *HaFT2* [46, 47] and the other in a gene with homology to a mitogen-activated protein kinase kinase kinase 14; one SNP on LG7 in a gene with high similarity to a gene in the armadillo repeat family of proteins in *A. thaliana*; one SNP on LG10 in the GRAS/DELLA transcription factor *GAI*; two SNPs on LG 12, one corresponding to an EF-hand-like domain-containing gene, and the other corresponding to a protein of unknown function; and one SNP located on LG 14 in a gene with high similarity to *Defective Cuticle Ridges* (*DCR*) in *A. thaliana*. BayeScan provided complementary outlier results by identifying three highly differentiated loci (SNPs within the *DCR* homolog, the GRAS/DELLA transcription factor, and the gene containing the EF-hand-like domain) already highlighted by Arlequin. Four loci had evidence of being significantly under-differentiated from both Arlequin and BayeScan. There were two under-differentiated loci on LG 13, including one SNP in a gene with an alpha-beta plait nucleotide binding role and another SNP in a gene with homology to 5′-AMP-activated protein kinase. SNPs in a glycoside hydrolase and a guanylate binding gene also had exceptionally low F_ST_, and were found on LGs 8 and 17, respectively (data not shown).

**Table 4:** Summary of candidate genes involved in local adaptation. F_ST_ values were determined by Arlequin and/or BayeScan and were cross-referenced against QTL information to determine the extent of QTL co-localization

Gene name	F_ST_	LG	cM Position	Arlequin ^a^	Bayescan ^a^	QTL ^b^
Mitogen activated protein kinase kinase kinase 14	0.38	6	53.72	2/5	0/5	A, B, C, D, E, F, G, H, N, O, X, Y, Z, AA
*DCR*	0.38	14	67.27	2/5	5/5	None
No annotated hit heliagene or NCBI	0.40	12	65.62	1/5	0/5	I, L, Y
*HaFT2*	0.47	6	65.9	2/5	0/5	E, F, H, M, N, O, T, U, V, W
Armadillo (ARM) repeat	0.36	7	19.29	1/5	0/5	D, E, J, L, P
No annotated hit heliagene or NCBI	0.37	4	73.86	1/5	0/5	J
GRAS/DELLA transcription factor	0.41	10	66.87	5/5	5/5	None
EF-hand-like domain	0.44	12	44.1	5/5	5/5	J, K, I, Q, R, S, Y

^a^ Fraction represents the number of times a particular locus was detected as an F_ST_ outlier

^b^ Letters represent co-localizing QTL. Key: A – Leaf shape [43], B – Number of ray flowers [32], C – Disc diameter [42], D – Height [32], E – Days to flower [32], F – Leaf fungal damage [44], G – Achene width [32], H – Days to flower [42], I – Leaf shape [32], J – Leaf number [44], K – Head total [44], L – Number of heads [32], M – Seed total [44], N – Leaf herbivory [44], O – Head clipping weevil [44], P – Head herbivory [44], Q – Branch number [44], R – Stem diameter [44], S – Leaf shape [44], T – Days to flower [43], U – Height [43], V – Leaf number [43], W – Leaf moisture content [43], X – Height [42], Y – Heads per branch [32], Z – Stem diameter [32], AA – Achene weight [32]

### Co-localization of SNP outliers with known QTL

The locations of our eight over-differentiated loci were compared to the locations of previously mapped sunflower QTL to identify traits potentially involved in local adaptation. On LG 4, an unannotated gene co-localized with a QTL for leaf number [44]. As noted above the distal end of LG 6 contains two F_ST_ outliers: *HaFT2* and a gene with a putative kinase function. Both of these co-localize with QTL related to flowering time in two sunflower mapping populations, ANN1238 × CMS 89 [32] and ANN1238 × Hopi [42]. This genomic region is actually known to contain multiple *HaFT* paralogs, including *HaFT1*, which has been shown to be important with respect to cultivated sunflower’s photoperiod response [46, 47]. In addition to co-localization with the flowering time QTL in this region, there are QTL for morphological traits (e.g., achene width, plant height, and number of ray flowers) and even a QTL for leaf fungal damage. The SNP outlier on LG 7, from an EST with homology to an ARM repeat protein, co-localizes with QTL for flowering time, plant height, leaf number, and head herbivory, as well [32, 44]. Interestingly, two loci with strong support from both Arlequin and BayeScan (the GRAS/DELLA transcription factor and the *DCR* homolog, which map to LGs 10 and 14, respectively), did not co-localize with any known QTL. One of the two outliers on LG12, an unannotated gene, co-localized with leaf shape and number of heads [32]. Finally, the EF-hand-domain containing gene co-localized with a QTL for head total (one way of describing the degree of branching), as well as leaf and branch traits, found on LG 12 (Table 4).

Under-differentiated loci co-localized with QTL for a variety of different traits. Of particular interest were two low F_ST_ outliers located near each other on LG 13 that co-localized with a shared set of QTL that included: number of branches, number of heads, head and leaf herbivory, stem diameter, achene length, leaf area, and stem height [32, 42–44].

## Discussion

Populations across the range of wild sunflower harbor an exceptional amount of phenotypic diversity. The extent to which those traits contribute to local adaptation is an important question that can be addressed in a number of ways including reciprocal transplants, common garden measurements, and population genome scans. In our analyses, many traits (e.g., flowering time, plant height, plant architecture, and seed oil composition) were differentiated in conjunction with latitude. As sunflower is a seed oil crop, there has been a considerable of research done to describe and uncover the genetic mechanism behind seed oil variation. In breeding lines, strong artificial selection has created divergent germplasm groups with vastly different oil profiles. In the wild, natural selection may act as a strong force in affecting what relative amounts of saturated and unsaturated fatty acids are most beneficial for populations living in certain environments.

### Common garden phenotypic variation

Seed oil composition exhibited significant latitudinal differentiation across the range. Previous studies of seed oil composition in a variety of species have revealed a negative correlation between saturated fatty acid content and latitude and degree of saturation at a relatively coarse geographic scale [45]. By quantifying the percentage of saturated fatty acids across the range of sunflower, we were able to identify a similar trend (Table 2; Additional file 1), albeit at a finer geographic scale. Given that these plants were grown in a common garden, we can infer that the observed differences have a genetic basis, and that functional polymorphisms in the oil biosynthetic pathway exist across the range of wild sunflower. The percentage of saturated fatty acids in seed oils is of considerable evolutionary importance with respect to germination under different environmental conditions. Saturated fatty acids are known to store more usable energy per carbon as compared to unsaturated fatty acids [45], but saturated fatty acids also have higher melting points than unsaturated fatty acids; the associated energy is thus less accessible in cooler temperatures. The resulting inference is that the production of unsaturated fatty acids in higher latitudes is advantageous because it ensures energy availability at lower temperatures [45]. Conversely, saturated fatty acids are better in lower latitudes because they are more energy rich while still being available to germinating seeds due to the comparably warmers temperatures.

Observed differences in flowering time can be interpreted in a similar framework. Growing seasons tend to be shorter in higher latitudes; thus, there is a premium on flowering early to allow seed set before the end of the growing season. Alternatively, in lower latitudes, there is typically a longer growing season that may select for later flowering plants that may grow to a larger size and produce more and/or higher quality seeds. It must, however, be noted that plant height and flowering time are developmentally correlated; as such, they form a suite of inter-related traits [48, 49]. The differentiation seen in this study confirms some of the patterns of diversity documented by Blackman et al., [46], with northern populations flowering significantly earlier compared to southern populations when grown at 16 h days. While common garden approaches do isolate the effects of genotype on trait variation, it should be noted that approaches like this do preclude the study of genotype-by-environment (G × E) interactions. Reciprocal transplants across the range would thus be useful to further characterize the relevance of the aforementioned traits in local adaptation. While not the focus of this study, it should be noted that altitude is also a possible cause of differentiation in a suite of traits, as shown by Kooyers et al., [50].

### Population genetic structure

The STRUCTURE analysis of the full dataset revealed an overall north/south division in the natural range of wild sunflower, with a transitional zone occurring in the vicinity Nebraska and Wyoming. Previous sampling of *H. annuus* genetic diversity had hinted at a similar north/south division [51], and our analysis builds on this finding by increasing the marker density and sampling density within each population. Historically, this latitudinal transect has seen similar patterns of genetic differentiation. For example, using transplant gardens, McMillian [52] showed that multiple grassland species exhibited heritable differences in flowering time in which northern populations flowered significantly earlier. When further STRUCTURE analyses were performed on northern and southern subsets of individuals, it was discovered that hierarchical structure exists in our dataset. In other words, the large north/south split identified in the full dataset may have obscured more subtle patterns that differentiate individual populations.

### Candidate adaptive loci

In terms of population genetic differentiation, we identified interesting possible candidates for conferring local adaptation with respect to flowering time. We found two outlier loci on chromosome 6 with SNPs that co-localize with a gene with putative kinase activity and *HaFT2*. Both loci co-localize with previously identified QTL for flowering time, [32, 42] in addition to other traits (Table 4). *FT2* is a gene whose *Arabidopsis* homolog has been shown to play a major role in promoting flowering [53]. Moreover, the region of sunflower LG 6 where this gene resides has been previously shown to influence flowering time in domesticated vs. wild sunflower [32, 42, 47]. It should be noted that the mapping parents for these crosses consisted of a wild × crop and wild × landrace. The extent of linkage disequilibrium (LD) of this region is currently unknown, although previous work indicates that, on average, LD decays quickly in wild sunflower [54]. Studies of cultivated germplasm suggested that there is variation in LD across the sunflower genome [55]. In addition to mapping information, *HaFT2* is an exceptional candidate for local adaptation due to previous gene expression work across the range of wild *H. annuus* [46]. In short days, a cline in gene expression was seen for *HaFT2* in which northern individuals exhibited higher expression than southern individuals, consistent with this gene playing a role in adaptive differentiation [46]. Our results add to the observation that *HaFT2* exhibits a latitudinal cline in gene expression that is consistent with the effects of selection by providing population genetic evidence of selection on this gene, as well.

We uncovered SNPs with significantly elevated population differentiation values on other chromosomes. A strongly differentiated SNP on LG 14 resides in the sunflower homolog of *Defective in Cuticle Ridges* (*DCR*). In *A. thaliana*, mutants of *DCR* have altered trichome development during leaf growth [56, 57]. Trichomes serve a multitude of functions in plants including: reflectance of sunlight to prevent damage [58], retention of water [59], and defense [60]. As many of the aforementioned factors may correlate with growing season, it is difficult to draw any conclusions without additional data. We cannot conclude, for example, that the variants documented herein are in any way causal in nature. Rather, they provide us with a preliminary pool of candidate adaptive regions for further study. Furthermore, since we lack knowledge concerning the strength of linkage disequilibrium in these genomic regions, these SNPs may simply be linked to causal polymorphisms found in nearby genes.

These F_ST_ outliers form a list of possible candidate genes for future experiments. Importantly, the extent of linkage disequilibrium needs to be assessed in these genomic regions in order to determine the size of the region of elevated population structure. A possible explanation for the absence of co-localizing QTL for some SNPs is that no wild × wild mapping populations currently exist for sunflower. Alternatively, many subtle (trichome density or morphology) and biochemical phenotypes have not been measured and thus could not have co-localized with population differentiation. Marker density has become the main limitation in genome scan studies of local adaptation in natural populations [61]. The advent of high-throughput methods such as restriction site associated DNA sequencing (RAD-seq) and genotyping by sequencing (GBS) have allowed researchers to obtain both large numbers of markers and an even genomic distribution [62–64].

## Conclusions

In this study we used 246 loci to characterize the range-wide genetic diversity and structure of the wild progenitor of an economically important crop species. These markers clearly indicated a genetic disjunction between northern and southern populations that occurs around the 40° north latitude, with populations in Nebraska appearing to be admixed (Fig. 2). This study also generated multiple candidate genomic regions for local adaptation as defined by the extent of their population genetic differentiation. The extent to which these genomic intervals are associated with previous trait mapping experiments is also considered. These loci represent larger physical genomic intervals that will be the focus of future molecular evolutionary analyses, gene expression comparisons across the range, and field studies to further examine their putative role in local adaptation.

## Additional files

Additional file 1: Results of REML analysis of phenotype data. (XLSX 30 kb)
Additional file 2: STRUCTURE bar plot of southern regions. (PDF 51 kb)
Additional file 3: Delta *K* plot for southern STRUCTURE plot found in Additional file 2. (PDF 22 kb)
Additional file 4: STRUCTURE bar plot corresponding to *K* = 6 for the six populations within the southern two regions. (PDF 60 kb)
Additional file 5: STRUCTURE bar plot of northern regions. (PDF 34 kb)
Additional file 6: Delta K plot for northern STRUCTURE plot found in Additional file 5. (PDF 10 kb)

## Abbreviations

AMOVA: Analysis of molecular variance
G × E: Genotype by environment
GBS: Genotyping by Sequencing
H_O_: Observed heterozygosity
LD: Linkage disequilibrium
LG: Linkage group
NMR: Nuclear magnetic resonance
QTL: Quantitative trait locus
RAD-seq: Restriction site associated DNA sequencing
SNP: Single nucleotide polymorphism
uH_e_: Unbiased expected heterozygosity
