# Supplementary figures and images for: Range-wide phenotypic and genetic differentiation in wild sunflower
Source: BMC Plant Biol. 2016 Nov 10;16:249. doi: 10.1186/s12870-016-0937-7 (PMC5103407; doi:10.1186/s12870-016-0937-7)

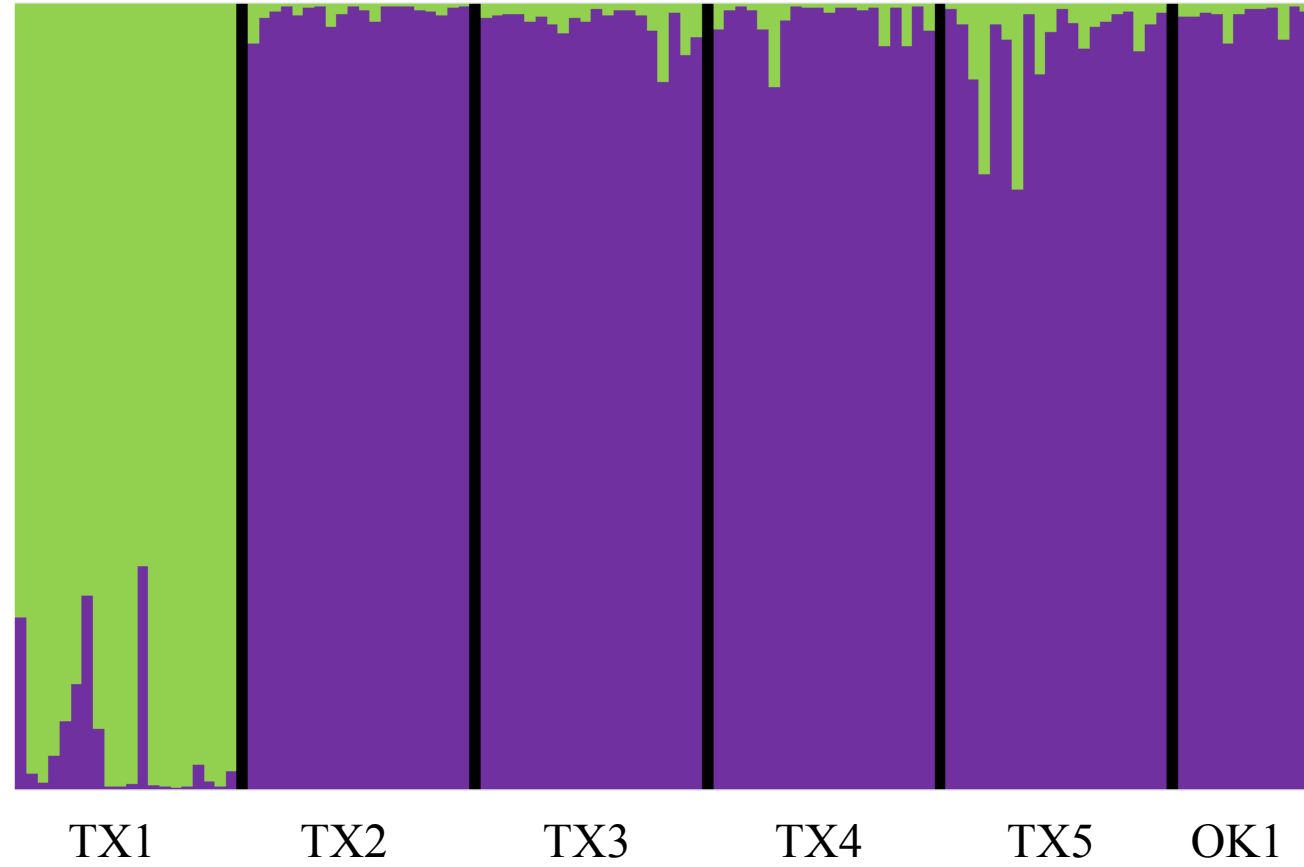

Supplement: Additional file 2: — STRUCTURE bar plot of southern regions. (PDF 51 kb) [file 12870_2016_937_MOESM2_ESM.pdf]

$$\text{Deltak} = \text{mean}(|L''(K)|) / \text{sd}(L(K))$$

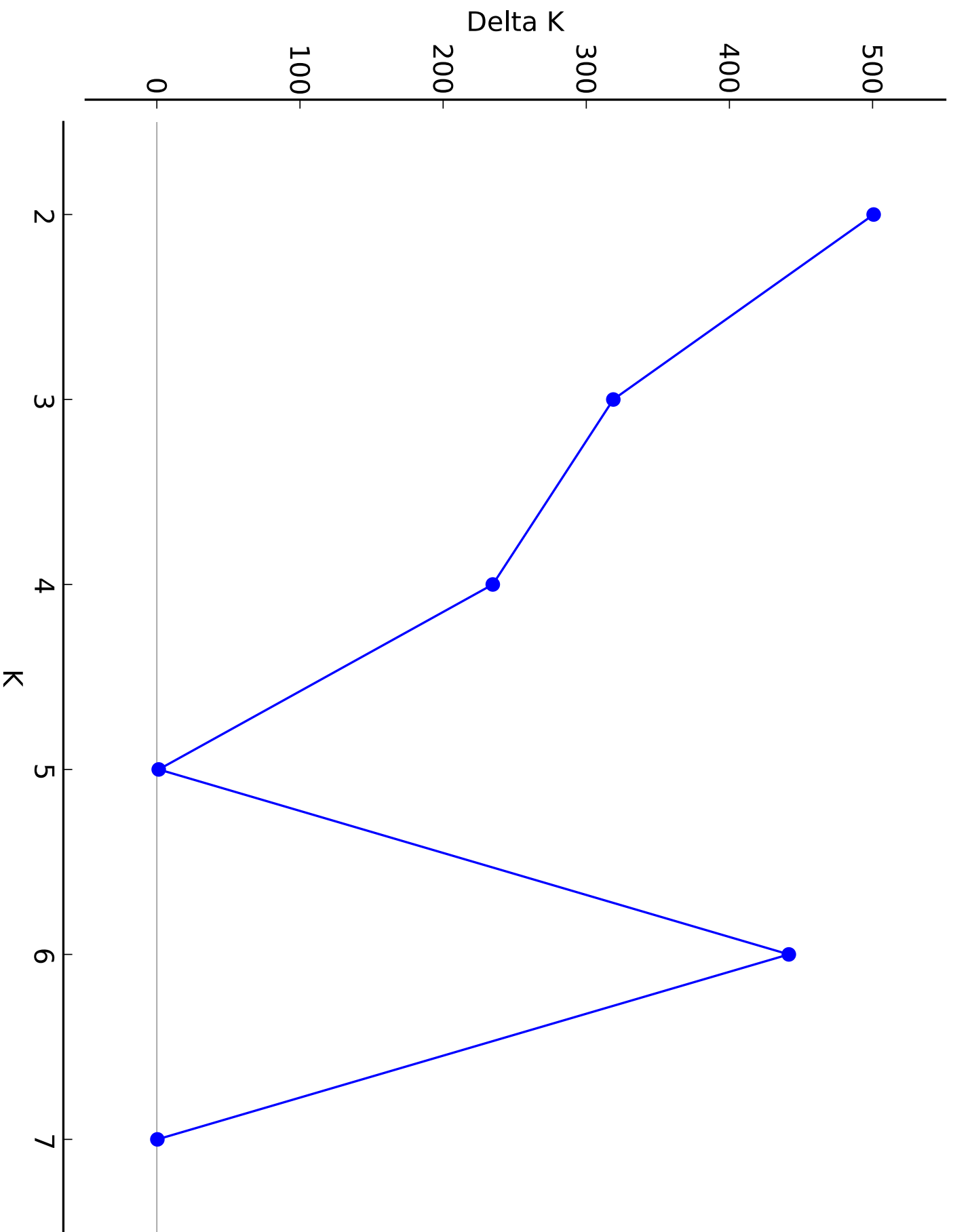

Supplement: Additional file 3: — Delta K plot for southern STRUCTURE plot found in Additional file 2. (PDF 22 kb) [file 12870_2016_937_MOESM3_ESM.pdf]

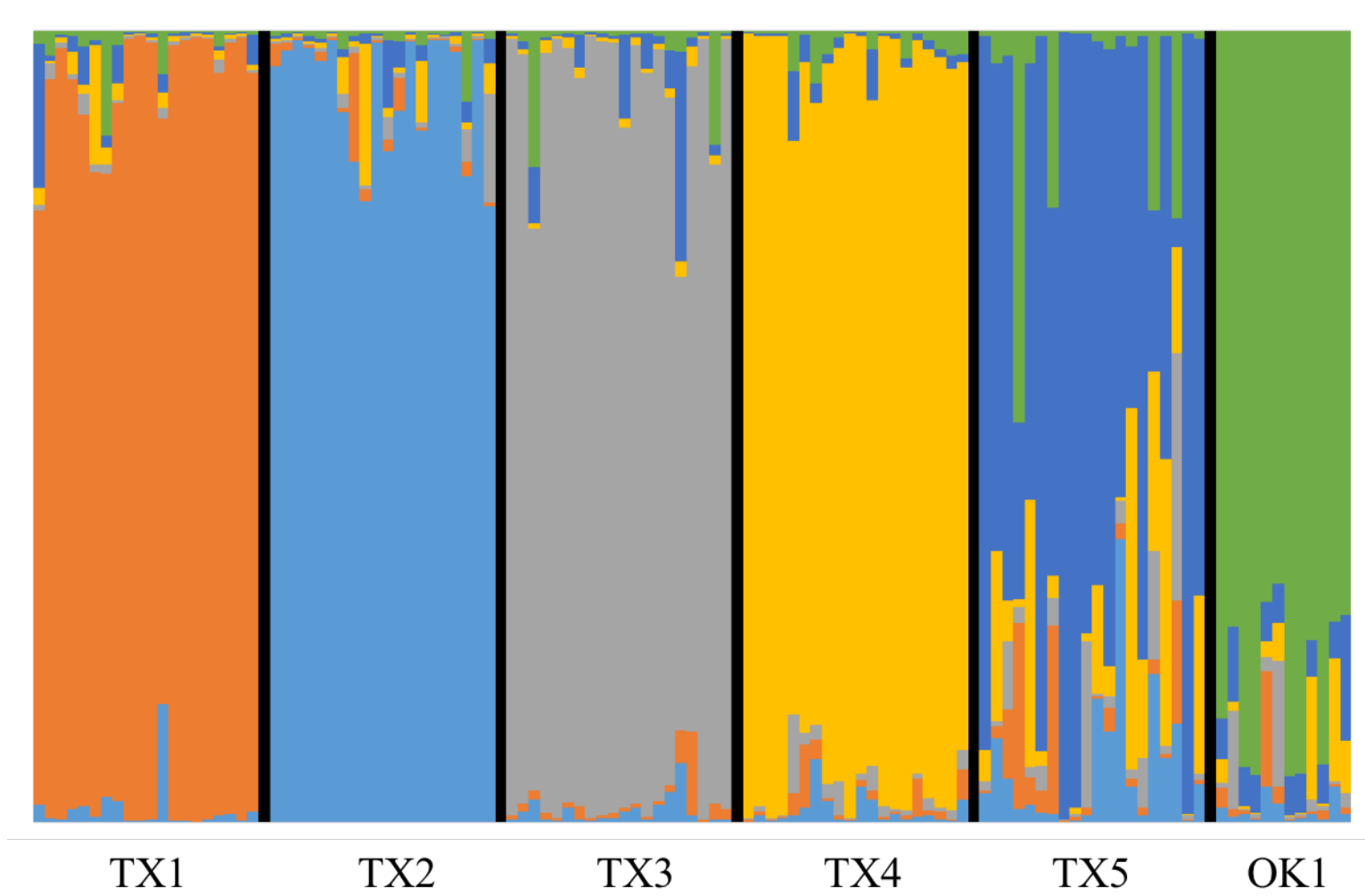

Supplement: Additional file 4: — STRUCTURE bar plot corresponding to K = 6 for the six populations within the southern two regions. (PDF 60 kb) [file 12870_2016_937_MOESM4_ESM.pdf]

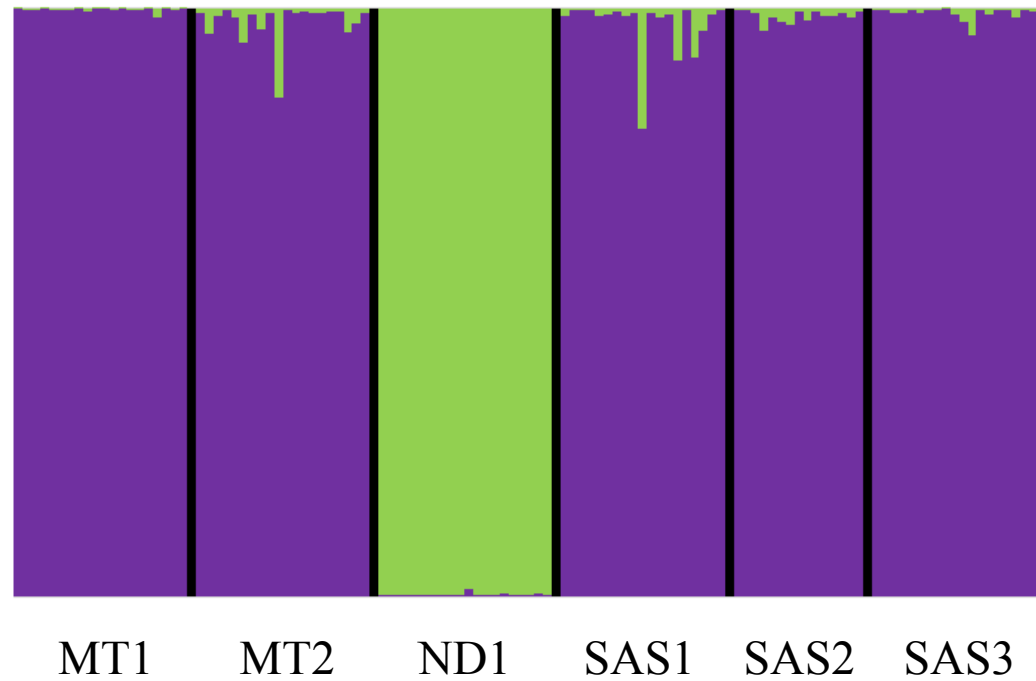

Supplement: Additional file 5: — STRUCTURE bar plot of northern regions. (PDF 34 kb) [file 12870_2016_937_MOESM5_ESM.pdf]

$$\text{DeltaK} = \text{mean}(|L''(K)|) / \text{sd}(L(K))$$

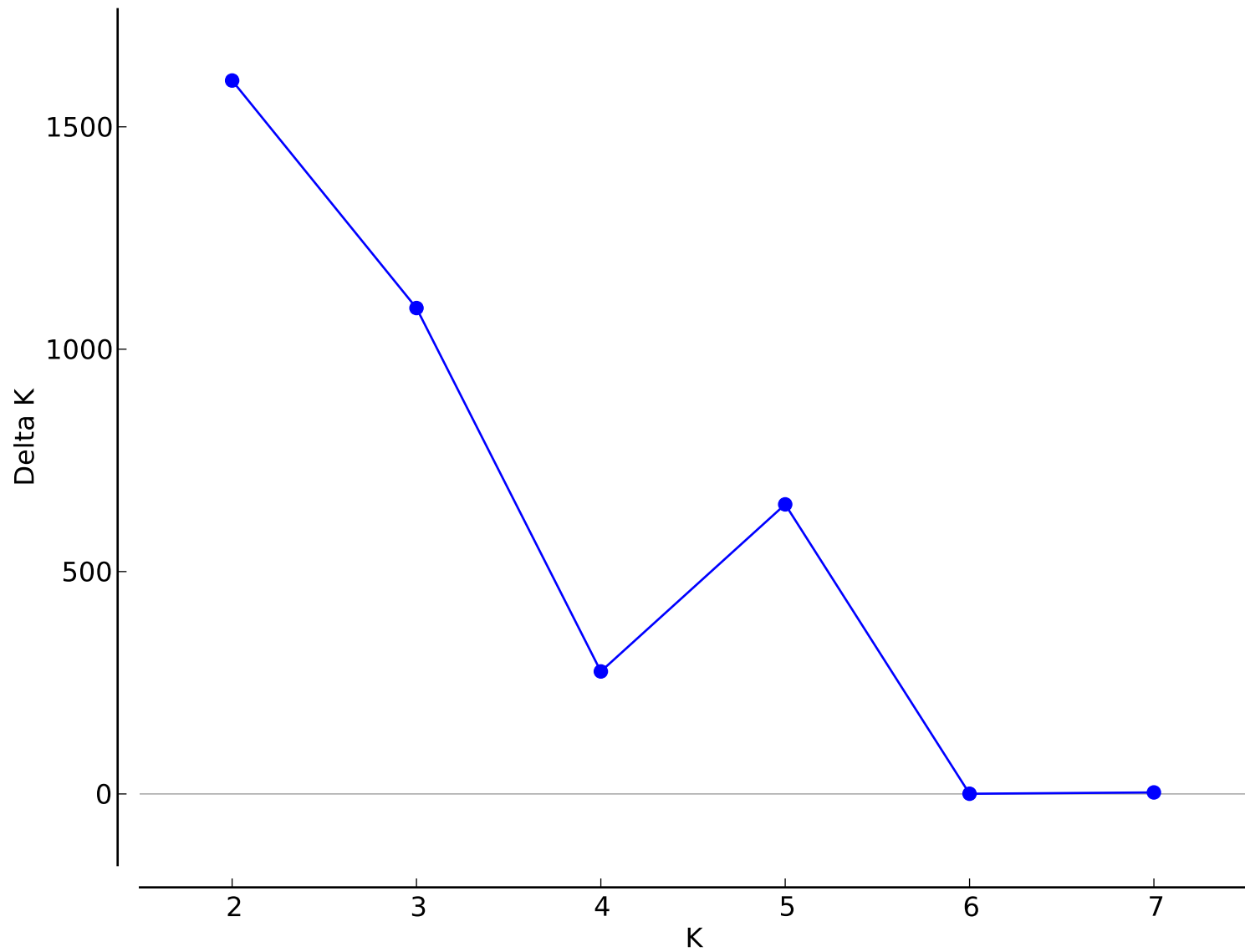

Supplement: Additional file 6: — Delta K plot for northern STRUCTURE plot found in Additional file 5. (PDF 10 kb) [file 12870_2016_937_MOESM6_ESM.pdf]
